# Supplementary material for: Impact of Inflammatory Burden on Voriconazole Exposure in Oncohematological Pediatric Patients Receiving Antifungal Prophylaxis after Allogeneic HCT
Source: Microorganisms. 2024 Jul 9;12(7):1388. doi: 10.3390/microorganisms12071388 (PMC11278995; doi:10.3390/microorganisms12071388)

## Supplementary Materials

**Supplementary Table S1.** Comparison of serum inflammatory biomarkers levels during episodes of voriconazole overexposure and those in therapeutic range and/or underexposure.

| **Serum Inflammatory Biomarkers Level [Median (IQR)]** | **Voriconazole Overexposure (N=163)** | **Voriconazole Non-Overexposure (N=467)** | ***p* Value** |
|---|---|---|---|
| C-reactive protein (mg/dL) | 9.30 (1.03-19.29) | 1.14 (0.35-4.38) | <0.0001 |
| Procalcitonin (ng/mL) | 3.0 (0.5-7.2) | 0.5 (0.2-1.2) | <0.001 |
| Interleukin-6 (pg/mL) | 209.6 (66.4-788.8) | 27.9 (8.9-89.9) | <0.0001 |

**Supplementary Figure S1.** Details of causes of inflammation and specific onset time for each included patient. HCT: hematopoietic stem cell transplantation.

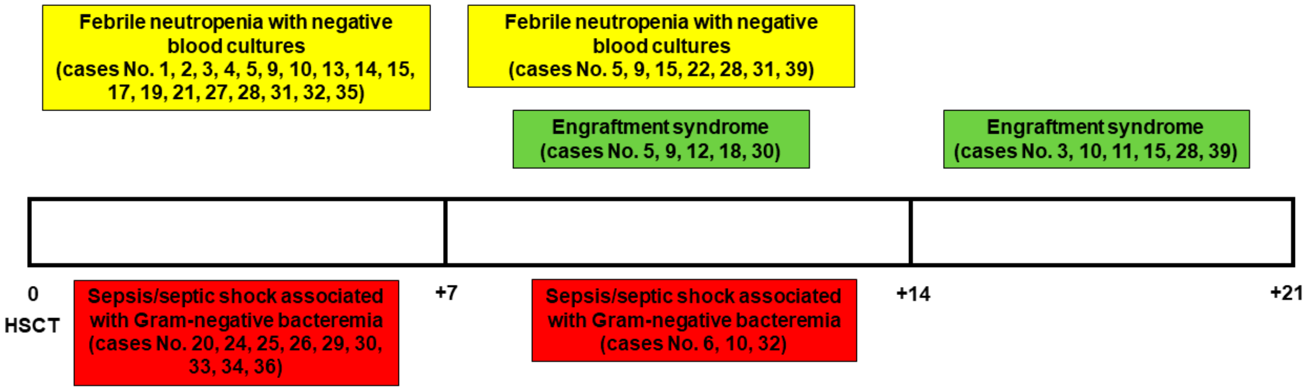

Supplement: Supplementary file 1 [file microorganisms-12-01388-s001.zip › microorganisms-3092454-supplementary.pdf]
